# The quality of family relationships in dementia: Mixed methods to unravel mixed feelings

**DOI:** 10.1177/14713012231220759

**Published:** 2023-12-15

**Authors:** Maria J. Marques, Manuel Gonçalves-Pereira, Marjolein de Vugt, Frans Verhey, Bob Woods, The Actifcare Consortium

**Affiliations:** CHRC, NOVA Medical School, Faculdade de Ciências Médicas, 50106 Universidade NOVA de Lisboa, Lisbon, Portugal; CHRC, NOVA National School of Public Health, 50106 Universidade NOVA de Lisboa, Lisbon, Portugal; CHRC, NOVA Medical School, Faculdade de Ciências Médicas, 50106 Universidade NOVA de Lisboa, Lisbon, Portugal; Department of Psychiatry and Neuropsychology and Alzheimer Centre Limburg, School for Mental Health and Neurosciences, 5211 Maastricht University, Maastricht, The Netherlands; Dementia Services Development Centre (DSDC) Wales, School of Medical & Health Sciences, 1506 Bangor University, Gwynedd, UK

**Keywords:** Alzheimer’s disease, dementia, quality of life, relationship quality, family care, dyadic perspective

## Abstract

**Objective:** Close relationships influence health and quality of life outcomes for people with dementia and their families. Yet, we know little on the role of different relationship domains with quantitative methods having proved to have limitations in this research field. We aimed to study these relationship domains over time, contrasting the views of people with dementia and their family carers, making use of both quantitative and qualitative approaches.

**Methods:** A convergent mixed methods design was adopted, analysing longitudinal data (four time points over three years) from 66 dyads of Portuguese community-dwelling people with dementia and their primary carers, from the EU-Actifcare project sample. Quantitative assessments used sociodemographic and clinical variables, and Positive Affect Index scores, with descriptive and inferential analyses. Qualitative data, collected through individual and joint semi-structured interviews, were explored using thematic analysis.

**Results:** Both quantitative and qualitative findings demonstrated that some domains of relationship quality are affected in different ways, with changes occurring at different stages. Some (e.g., ‘communication’) may even improve after initial decline. ‘Closeness’ was consistently altered over time, from carers’ perspectives, and played an important protective role regarding institutionalisation. Overall, changes in the relationship quality were perceived differently by people with dementia and their carers, and these divergent perspectives often led to tension. Qualitative data revealed that ‘mixed feelings’ (ambivalence) involve complex experiences, arguably more difficult to manage than negative feelings alone. Furthermore, perceived informal support, particularly from the extended family, and receiving formal services’ assistance, seemed to facilitate positive (re)appraisals of the relationship.

**Conclusions:** A deeper understanding of relationship quality and its domains as dementia progresses may help tailoring interventions to tackle modifiable aspects of relationships, meeting the needs and cherishing the resources of dyads and families. Timely assessments could identify relationships at risk and need for support, including for alternative caring arrangements.

## Introduction

Dementia is a leading cause of disability and dependency among older people (Livingston et al., 2020). Adverse outcomes include cognitive and functional decline, so-called behavioural and psychological symptoms of dementia, reduced quality of life, institutionalisation and death (Fauth et al., 2012; Miller et al., 2019; Norton et al., 2009; O’Shea et al., 2020; Spitznagel et al., 2006; Woods et al., 2014). Informal carers (mainly family and friends) are at risk of burden, psychological distress, somatic complications and diminished quality of life (Cheng, 2017; Lindeza et al., 2020).

The availability and quality of close relationships may protect against these adverse outcomes (Ablitt et al., 2009; Edwards et al., 2018; Quinn et al., 2009). ‘Relationship quality’ is defined as the subjective global evaluation of the relationship between the person with dementia and their informal carer (i.e. how happy an individual is in their relationship) (Fincham & Rogge, 2010). Evidence on the benefits of psychosocial interventions in dementia is growing (Cheng et al., 2019; Livingston et al., 2020; McDermott et al., 2019; Oyebode & Parveen, 2019), particularly of dyadic interventions (Laver et al., 2017; Poon, 2019; Van’t Leven et al., 2013). To assume a more positive ‘couple-centred’ tone, these interventions should move beyond a narrow focus on behavioural ‘control’ (‘management’) to encompass wider aspects of life, including relationship quality (Oyebode & Parveen, 2019; Stedje et al., 2023). Maintaining or improving dyadic relationships promotes quality of life (Clare et al., 2014; Woods et al., 2014) and social health in dementia (Dröes et al., 2017). It may also help regarding behavioural and psychological symptoms and even cognitive and functional decline (Tschanz et al., 2013). Carers’ positive outcomes include less reactivity to ‘challenging’ behaviours (Steadman et al., 2007), acknowledging positive aspects of caregiving, mitigating burden and distress and shaping positive attitudes towards formal care (Quinn et al., 2012; Steadman et al., 2007; Tough et al., 2017). Overall, this might lower informal care costs (Rattinger et al., 2016), and delay institutionalisation (Spruytte et al., 2001).

Overall, there is now a body of evidence from family caregiving research in dementia that also supports the well-established concepts of person-centred care and relationship-centred care (Kitwood & Brooker, 2019), together with the need to develop environments which enhance interpersonal aspects (Bielsten & Hellström, 2019a; Gilbert et al., 2023). Notwithstanding, some shortcomings are still noteworthy (Stedje et al., 2023) and more research is needed on relationship quality. First, only a few studies contrasted the perspectives of people with dementia and their family carers’ (Marques et al., 2021; Mortazavizadeh et al., 2020; Spector et al., 2016; Woods, 2009; Woods et al., 2012; Wright, 1991). People with dementia generally rate relationship quality higher than carers (Clare et al., 2012; Rippon et al., 2020; Spector et al., 2016; Woods, 2009; Wright, 1991). What contributes to differences between the two perspectives remains unclear, but carer’s stress (more than lack of awareness of the person with dementia) may be the main predictor of the discrepancy (Clare et al., 2012; Marques et al., 2019; Mortazavizadeh et al., 2020; Pfeifer et al., 2013; Woods, 2009).

Next, evidence on the time course of relationship quality is particularly sparse. In a longitudinal study we examined the trajectories of relationship quality and its influencing factors in a large European cohort, and found that caregiving negatively affected relationship quality over 12 months (Marques et al., 2021).

Finally, we know little about which domains of relationship quality are more important, and how they affect outcomes in dementia (Edwards et al., 2018).

Recently, a narrative synthesis systematic review addressed this, albeit specifically in spousal relationships (Stedje et al., 2023). The authors reviewed 39 studies and grouped twenty influencing factors into two main themes (*the world of us* and *the world outside of us*) and six factors (attitudes and strategies, behaviour and activities, emotional connectedness, activities and experiences outside home, social behaviour and roles, belonging and safety). Still, they called for further research to understand those factors, their relations and cultural context (Stedje et al., 2023), not least because studies on non-spousal caregiving relationships were not reviewed.

Although much research explored the ‘communication’ domain and how it may be preserved or affected (Bruinsma et al., 2020; Clare et al., 2012; de Vugt et al., 2003; Feast et al., 2021; Marques et al., 2019) we lack robust evidence on the impact of dementia on other aspects of relationship quality (e.g. ‘closeness’, ‘similar views’, ‘shared activities’). Most studies even consider relationship quality as unidimensional, using exclusively quantitative approaches with brief self-report measures, scoring from ‘poor’ to ‘excellent’, over-looking the complexities of multi-faceted relationships in real-life (Fincham & Rogge, 2010). Meanwhile, the important qualitative explorations pinpointed that positive and negative relationship appraisals coexist (Wadham et al., 2016). However, most qualitative approaches were cross-sectional, involved a small number of participants, or focused mainly on spousal relationships, despite an increasing interest in other types of family relationships (Bielsten & Hellström, 2019b; La Fontaine & Oyebode, 2014). Notably, quantitative and qualitative methods were seldom combined. This hinders a deeper understanding of relationships: for instance, ambivalent feelings, that is experiencing conflicting emotions at the same time – positive and negative feelings – could be unveiled (Losada et al., 2017). Therefore, despite growing evidence on relationship quality and its determinants or outcomes (Edwards et al., 2018; Stedje et al., 2023), we need insights on how the different elements of relationship quality might influence dementia caregiving, including ambivalent experiences.

The evidence gap calls for mixed methods designs providing a fuller picture that can bring together description, quantification and phenomenological understanding of relationship quality, namely the individual and dyadic characteristics and changing circumstances over the course of dementia. On the one hand, we should involve the two members of the dyad as active participants in the research and encourage in-depth explorations of relational aspects. On the other hand, standardized assessments remain useful, particularly when planning longitudinal follow-ups. For instance, the Positive Affect Index (PAI) (Bengston & Schrader, 1982) provides subjective evaluations of a dyad’s relationship quality (intrapersonal approach) over different domains.

In the present study, we aim to determine which elements of the relationship quality are affected, and to what extent, and to contrast the perspectives of people with dementia and family carers regarding changes in the quality of their relationship, over an extended follow-up. Additionally, we explore how participant views on relationship quality, as based on quantitative results and qualitative findings, converge or diverge.

## Methods

### Design

This study is drawn from the Actifcare (ACcess to TImely Formal Care) EU-JPND project in eight countries (Germany, Ireland, Italy, Netherlands, Norway, Portugal, Sweden, United Kingdom), which included a one-year prospective study of 451 dyads of people with mild-to-moderate dementia and their family carers (Kerpershoek et al., 2016).

Here, we used longitudinal data from the Portuguese cohort, extending assessments through a 36-month follow-up and focusing on relationship quality analyses.

A parallel-databases variant of the convergent mixed methods design allowed us to obtain different but complementary quantitative and qualitative data from the same participants over a three-year period. Quantitative and qualitative strands were collected and analysed independently, and brought together during interpretation (Creswell & Creswell, 2018; Ivankova et al., 2006). Both strands were implemented at the same time-point by the same researchers and given equal emphasis within the study.

### Participants

We studied 66 dyads (*n* = 132 community-dwelling participants), recruited from settings including general practices, memory clinics, and Alzheimer Portugal’s affiliates (Gonçalves-Pereira et al., 2019). The diagnosis of people with dementia followed Diagnostic and Statistical Manual of Mental Disorders (DSM-IV) criteria, with a Clinical Dementia Rating (CDR) (Morris, 1993) of mild to moderate stage. Exclusion criteria included receiving significant (personal) care from formal services at baseline on account of dementia. The EU-Actifcare protocol is detailed elsewhere (Kerpershoek et al., 2016).

### Procedures

Participants were assessed on entry, and again at 6, 12 and 36 months. They were seen at home, unless preferring elsewhere. Regarding quantitative assessments, one to two visits of up to two hours were required. Regarding qualitative data, we adopted a multi-level semi-structured interview approach (Blake et al., 2021) wherein dyads were interviewed first jointly and then individually. Eventually, both were brought back together for a 10-min session that enabled debriefing and ending-up in a positive atmosphere. Interviews were conducted by the first author through one and a half to two hours, plus half an hour for each joint dyad.

Approval for the study was granted by the Portuguese Data Protection Authority (CNPD), the Ethics Committees of NOVA Medical School and clinical services involved. All participants provided written informed consent.

### Measures

In the quantitative arm, participants completed questionnaires on sociodemographic and clinical-functional variables (Kerpershoek et al., 2016). The PAI (Bengston & Schrader, 1982) assessed relationship quality and was rated separately by people with dementia and carers. This 5-item scale measures current relationship quality in five domains (closeness, communication, similar views, shared activities, generally getting along). Responses are rated on a 6-point scale from 1 (not well) to 6 (extremely well), with a total from 5 to 30 (higher scores reflecting better relationship quality). It has been used with people with dementia (Clare et al., 2012), showing good internal consistency (Cronbach α .81) and reasonable test-retest reliability (*r* = .66) (Woods, 2009). In the present study, Cronbach’s α_s_ were .82 (people with dementia) and .83 (carers).

Regarding qualitative data, semi-structured interviews were conducted to explore relationship quality from each participant’s perspective (e.g. ‘how would you describe your current relationship with your relative?’). People with dementia and carers described relationship quality in their own words, and (at follow-ups) reported changes in its different domains. Interviews also covered existing support from social and healthcare professionals, other significant others, and the mixture of challenges and positive aspects associated with dementia.

### Analysis

For quantitative data, descriptive and inferential analyses were employed, using Pearson correlation coefficients, independent-sample t-tests and repeated measures Anova. The significance threshold was set at .05. We used the Statistical Package for the Social Sciences (SPSS) for Windows version 25®, considering only participants who completed all four PAI assessments (baseline to 36 months).

For qualitative data, a reflexive thematic analysis was used (Braun & Clarke, 2012, 2021). Interviews were recorded, transcribed by the first author, and entered into NVivo12® for coding. The first author analysed all transcripts using Braun and Clarke’s six-step thematic analysis process: data familiarisation; initial coding (brief descriptive or interpretive labels useful in addressing the aims of the research); generating themes (shared meanings in relation to a particular topic or question) and sub-themes; reviewing potential themes; defining and naming themes; summarisation. The initial coding and theme development was flexible and evolved throughout the analytical process. The internal homogeneity of the themes (i.e. making sure everything within a theme is similar) was mainly led by the first author. The external homogeneity (among themes) involved the two co-authors MJM and MGP, who advised on the coding scheme and the most suitable solution (e.g. two candidate sub-themes were merged to produce the sub-theme ‘mixed-feelings’). The refining of themes was thoroughly documented. There was no predefined framework for coding themes.

## Results

Quantitative results are summarised in tables. Qualitative findings are presented with anonymised quotations from individual or joint interviews, illustrating identified themes and subthemes.

Table 1 depicts participants’ demographic and clinical characteristics, also detailed elsewhere (Gonçalves-Pereira et al., 2019).

**Table 1.** Baseline sociodemographic and clinical characteristics of people with dementia and their carers.

	Participants (*n* = 66 dyads)
People with dementia (*n* = 66)	
Age, years, mean (SD, range)	77.2 (6.2, range 57–91)
Sex, women *n* (%)	41 (62.1)
Education, years, mean (SD)	6.4 (6.1)
Living alone, *n* (%)	2 (3.0)
Type of dementia, *n* (%)	
Alzheimer’s type	25 (37.9)
Vascular	8 (12.1)
Mixed (Alzheimer’s and vascular)	7 (10.6)
Lewy body	2 (3.0)
Other	4 (6.1)
Not known	20 (30.3)
Cognitive impairment (MMSE)	17.8 (4.8)
Dementia severity (overall CDR)	1.1 (.3)
Neuropsychiatric symptoms (NPI-Q), mean (SD)	6.8 (5.5)
IADL function, mean (SD)	3.7 (2.0)
Basic ADL function (PSMS), mean (SD)	3.7 (2.0)
Relationship quality (PAI)	22.0 (4.3)
Carers (*n* = 66)	
Age, years, mean (SD, range)	64.89 (15.1, range 35–92)
Sex, women *n* (%)	44 (66.7)
Education, years, mean (SD)	9.0 (6.3)
Relationship to the person with dementia, *n* (%)	
Spouse/partner	40 (60.6)
Adult children	20 (30.3)
Other (e.g. son/daughter in law; sibling)	6 (9.1)
Anxiety (HADS), mean (SD)	6.5 (3.8)
Depression (HADS), mean (SD)	6.3 (4.3)
Stress (RSS), mean (SD)	22.2 (11.5)
Relationship quality (PAI)	21.2 (4.4)

CDR, Clinical Dementia Rating Scale; HADS, Hospital Anxiety and Depression Scale; IADL, Instrumental Activities of Daily Living; MMSE, Mini Mental State Examination; NPI-Q, Neuropsychiatric Inventory Questionnaire; PAI, Positive Affect Index; PSMS, Physical Self-Maintenance Scale; RSS, Relatives’ Stress Scale; SD, Standard Deviation.

*p ≤ .05 **p ≤ .01 ***p ≤ .001.

Fifty-eight dyads were reassessed at time point 1 (6 months), 54 at time point 2 (12 months), and 45 of these at time point 3 (36 months). Attrition was due to participant withdrawal/not willing to collaborate, stating exhaustion (6), carer or person with dementia health issues (4), moving abroad (1) institutionalisation (4) or death (6).

### Quantitative results

Relationship quality declined over time (Table 2). As assessed by carers, it declined between 6 months and 3-year (*p* = .001) and between 1 and 3-year assessments (*p* < .001) [F (3, 129) = 6.489 *p* = .003]. Ratings by people with dementia also declined (*p* = .026) between 6-month and three years assessments [*F* (3, 132) = 2.798, *p* = .053] (Table 2).

**Table 2.** Mean scores on the Relationship Quality (PAI) across all four points.

H	Baseline		T1 (6 months)		T2 (1-year)		T3 (3-year)		F
	M	SD	M	SD	M	SD	M	SD	
PAI (PwD)	21.67	4.64	22.29	4.31	21.47	3.55	20.82	3.04	2.978
PAI (CG)	20.61	4.62	20.91	4.40	20.27	4.26	18.95	3.65	6.489**

*p ≤ .05 **p ≤ .01 ***p ≤ .001.

Regarding changes on RQ domains over the three years, carers’ ratings declined in ‘closeness’ F(3, 132) = 4.202, *p* = .017, and ‘shared activities’ F(3, 132) = 9.332, *p* < .001. People with dementia ratings showed a marginally significant decline in ‘similarity of views’ F(3, 126) = 2.795, *p* = .065 and ‘generally getting along’, F (3, 135) = 2.446, *p* = .080 (Table 3).

**Table 3.** Mean ratings on relationship quality (PAI) individual items at baseline, follow-up 1 (6 months), follow-up 2 (1-year) and follow-up 3 (3-year).

Item	People with dementia		Carers		t	Sig
	M	SD	M	SD		
Baseline						
Closeness	4.74	1.01	5.02	.98	−1.936	.057
Communication	4.05	1.05	3.77	1.16	1.680	.098
Similarity of views	4.03	1.06	3.32	1.39	3.389	.001***
Shared activities	4.52	1.28	4.56	1.22	−.247	.805
Generally getting along	4.61	.99	4.58	1.00	.222	.825
6 months						
Closeness	4.87	1.09	4.98	1.06	−.735	.465
Communication	4.09	.90	3.70	1.16	2.508	.015**
Similarity of views	3.98	1.24	3.20	1.34	3.577	.001***
Shared activities	4.71	1.15	4.80	1.30	−.485	.630
Generally getting along	4.71	.99	4.47	1.05	1.423	.160
1 year						
Closeness	4.76	1.05	4.82	1.15	−.434	.666
Communication	4.04	.87	3.59	.98	2.568	.014*
Similarity of views	3.84	.81	3.02	1.21	3.965	.000***
Shared activities	4.54	.99	4.58	1.18	−.221	.826
Generally getting along	4.49	1.00	4.39	1.06	.647	.521
3 years						
Closeness	4.64	.91	4.49	.97	.980	.333
Communication	3.84	.64	3.47	.89	2.576	.013*
Similarity of views	3.58	.72	2.89	1.11	3.572	.001***
Shared activities	4.36	.74	3.96	.90	2.404	.020*
Generally getting along	4.4	.78	4.20	.94	1.244	.220

*p ≤ .05 **p ≤ .01 ***p ≤ .001.

At baseline, people with dementia rated ‘similarity of views’ higher than carers t (64) = 3.389, *p* = .001. At 3-year, people with dementia ratings were higher than carers’ for ‘communication’ t (45) = 2.576, *p* = .013, ‘similarity of views’ t (43) = 3.572, *p* = .001 and ‘shared activities’ t (43) = 2.404, *p* = .020, but did not diverge significantly for ‘closeness’ and ‘generally getting along’ (Table 3).

### Qualitative results

**Figure 1.**
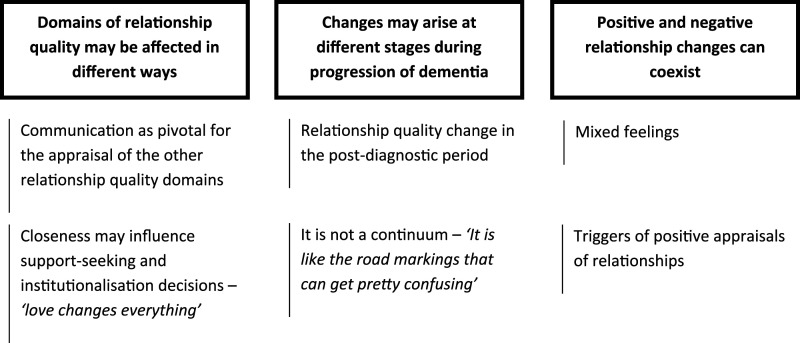
Themes and sub-themes developed through the thematic analysis.

The thematic analysis resulted in a final structure of three themes, with two subthemes each, incorporating person with dementia and carers’ perspectives (Figure 1).

## Domains of relationship quality may be affected in different ways

### Communication as pivotal for the appraisal of the other relationship quality domains

For most carers, adult-children or spouse/partner of the person with dementia, communication breakdown was perceived as a main challenge to the relationship, contributing to psychological distress. Several carers regretted using harsh forms of communication.

I feel like I’m always on the lookout as a lifeguard. But in this case, I can’t save the person I love. Look, I do know I must be patient. But sometimes I say: ‘Shut up, I can’t hear you no more’, especially when I’m too tired or worried. And then I get so frustrated with my attitude’. (spouse)

Communication problems increased with lack of spontaneous speech or response from the person with dementia (apathy) or behaviours as agitation or aggressiveness. Some carers also shared how dysphasia could contribute to difficulties engaging in conversations.

Communication became difficult. She doesn’t look at me when I speak. She keeps staring out into empty space. There is no conversation, not even about unimportant, foolish things. It’s like living with a dead person… nerve wracking, indeed. (adult child)

It is like they get so stressed out and cannot express it in any way other than being verbally aggressive. Sometimes he even throws things and curses. He never used to curse. And I lose my temper. (spouse)

She feels embarrassed and doesn’t even want to try. She used to be a teacher, you know… I think she feels incompetent due to her language difficulties. (spouse)

These communication issues hindered some carers from sharing their thoughts and feelings with their relative with dementia, affecting reciprocity and ‘closeness’. Apathy affected companionship: carers missed joint activities with their spouse/partners or parents. ‘Shared activities’ declined because the person with dementia was no longer interested, or the carer took no pleasure from activities with someone rarely responsive.

It is awful to be around with someone who doesn’t talk. He remains in his own little world and seems surprised when I speak to him. We used to talk for hours in the past… We did everything together, shopping, watching TV, walking…even washing the dishes. I do miss that… (spouse)

Contrarily, a few carers reported positive changes in ‘communication’, increasing reciprocity and ‘closeness’.

She talks more about the past, now. And I got to know more about my own past, feeling closer to my mum. (adult child)

During individual interviews, most people with dementia evoked carers’ efforts to empathize, allowing them to feel heard and valued. Alternatively, feeling patronized or pressured to give ‘correct’ responses discouraged them from continuing with the conversations.

It’s nice when he listens to me instead of telling me to shut up. (person with dementia)

I don’t like when my daughter tries to finish my sentences. If she could just listen… She is always rushing me because I can’t reply or speak fast enough. (person with dementia)

Participating in ‘shared activities’ was pivotal for meaningful communication. A few participants with dementia emphasized seeking opportunities to interact and maintain proximity, by joining activities they observed their carer doing.

You’ve got to find common interests… Something to talk about … He (husband) joined a walking group. And I’m stuck on this couch doing nothing… I usually say, ‘why don’t you take me?’. (person with dementia).

Joint interviews allowed some participants with dementia to communicate gratitude for their relatives’ support, giving their carers the opportunity to feel appreciated.

I am extremely grateful, obviously… (to the family) but this is very hard to say. It was always difficult for me to acknowledge the support received, from my relatives or others. (person with dementia)

It’s good to know he understands my efforts. He never told me that. (spouse)

### Closeness may influence support-seeking and institutionalisation decisions – *‘love changes everything’*

At baseline and 6-month interviews, some carers reported having felt emotionally closer to their relatives after dementia onset, because caregiving often implicates more frequent interactions.

Right after the diagnosis and initial symptoms, we’ve become even closer. Do you know that music, ‘Love changes everything’? My wife used to love it… Still does. There was a sense of urgency and wanting to seize every moment before the disease worsened. (spouse)

However, at one and three-year follow-ups, most carers considered that behaviours such as apathy and diminished joint activities compromised closeness and relationship quality overall. Changes resulted in isolation, feelings of rejection and loss, particularly when people with dementia could not recognise their carers any longer.

I miss doing things with my mother. As much as I try, she seems to have no interests. (adult child)

Some carers also reported that their relative’s role reversal, from care provider to care receiver, and their new responsibilities to manage care (not exactly nurturing, as previously), also compromised closeness.

It’s not that you don’t love him anymore. Your relationship changes from being a caring partner to being a caregiver… with no intimacy or affection. (spouse)

Over follow-ups, carers that requested formal community services (home or day care), or thought of institutionalising their relative, reported loss of emotional bonds and mutuality as main reasons. Other factors included people with dementia dependency, safety concerns, and behaviour issues or carers’ health.

Eventually, I just disconnected. Felt I was living with a dead person that was still alive. I had to apply for the nursing home. (adult child)

I always said that I would take care of my wife until the end. And, in the first years, I succeeded. But it’s harder to have a ‘stranger’ side by side, every minute. (spouse)

Some carers also expressed reluctance towards formal services, considering caregiving as a duty. In this context, guilt feelings emerged. Notwithstanding, all carers recognised the need for support, either for themselves or their relatives.

It was the hardest decision of my life (to institutionalise). And I feel an overwhelming guilt. (spouse)

How can I do that (place him in a nursing home)? He always took care of me… Even when I had cancer. But I can’t stand it no more. (spouse)

## Changes may arise at different stages during progression of dementia

### Relationship quality change in the post-diagnostic period

Most carers referred to dementia diagnosis, and the subsequent months, as a critical period in which too many adjustments were required, and coping strategies seemed less effective. Some reported a severe impact on relationship quality, namely regarding ‘communication’.

I already suspected for months, but it was right after diagnosis that I felt greater changes. I was still in shock and everything that I said or did only seemed to worsen things. I felt helpless… (spouse)

Most people with dementia also experienced distress in this period.

I was resentful and sad. And I couldn’t look at my wife. I felt I had let her down. I just wanted to die… (person with dementia)

Despite the importance of adjustment during initial stages, most carers received little timely support (including information about the condition or resources available, even less so regarding the potential impact on relationships).

I realised there is some information for those who know how to use computers…not my case. My husband always did that for me. We have few services and resources available where we live. More important, no one ever prepared me for the changes I would experience in my marriage. I was far from imagining… (spouse)

In the three-year follow-up interviews, most carers identified this post-diagnostic period as most distressful, not so much due to dementia process itself but to their loss of expectations.

Looking back, I was more distressed about the changes in our plans as a couple and my partner’s sadness than about the management of the disease itself. We were waiting for the retirement to finally move to our country house, travel and support our grandchildren. I was so angry then… (spouse)

### It is not a continuum – ‘It is like the road markings that can get pretty confusing’

Interviews’ findings over time showed that the relationship quality is dynamic: changes occurred at different stages of dementia, with setbacks and successes. For instance, at baseline, some carers mentioned going down a few steps in ‘communication’ but then no further reduction over one year. Most even mentioned improvement in communication strategies after this initial ‘‘turmoil’. Some participants with dementia also acknowledged the dynamic nature of their relationship: first they were concerned with being a burden but eventually learned to identify gratitude as crucial to reciprocity:

If we’d known that she had dementia, then you would have just accepted that she couldn’t remember. If somebody told us, we could have used a different approach with her. It is like the road markings that get pretty confusing. I learned to respond to her and guide her towards better decisions, as opposed to challenging her on her wrong decisions and doings. (adult child)

In the beginning, the idea of receiving support was very difficult for me. I didn’t want to be a burden. But time and specially these joint interviews helped me to understand that I would do the same for her, as she (spouse) has just said. I’m also thankful because you (the interviewer) are interested in listening to me. (person with dementia)

Some carers also highlighted ‘turning points’ associated with positive instances over the timeline (related with e.g., empathy, interaction, gratitude).

I thought ‘That (person with dementia) could be me!’. (spouse)

It’s the first time (during the joint interview) that I feel appreciated. That encourages me to hang on! (adult child)

A few carers voiced positive long-term changes, or described examples of personal growth despite cognitive decline and other dementia challenges.

You know…not everything is bad. He (husband) was always very formal and emotionally restrained. The military type…. My daughter even says that her father now expresses his love more openly. He even sings around the house. (spouse)

My mother started to paint and loves it. She was always a creative person but never had the chance or time to embrace it. I started to buy her those mandala books… (adult child)

## Positive and negative relationship changes can coexist

### Mixed feelings

During baseline and 1-year follow-up interviews, carers mainly emphasised negative aspects. In the three-year follow-up interviews, carers expressed negative and positive aspects, sometimes overtly ambivalent feelings. Simultaneous positive and negative feelings were more likely to coexist with: changes in relationship quality with progression of dementia; a difficult relationship before dementia onset; willingness to care but also to avoid excessive demands and preserve spare time. Female adult child carers reported ambivalence due to conflicting responsibilities, such as competing demands from relatives with dementia and spouses or children, or caregiving and professional duties. For some carers, these mixed emotions represented highly challenging experiences, more difficult to deal with than negative feelings alone.

It’s really hard. I can’t even share this with my relatives. To be honest, I have mixed feelings. I love and hate him, I feel all the emotions at the same time. There are moments when I’m barely sure of what I actually feel. (spouse)

Both at baseline and follow-ups, people with dementia were less inclined than carers to criticize aspects of their relationship.

I’m doing OK coping with it…but only because I’ve got her (wife) here all the time. (person with dementia)

Some participants with dementia reported a decline in similarity of views over the three years, fostering disagreements and conflicts. This difference in perspectives posed additional challenges to relationships.

She treats me like a child. We used to share views but now we disagree on everything. (person with dementia)

### Triggers of positive appraisals of relationships

Family resources, formal support, previous experiences with major health issues and good memories were underlined by most carers as facilitating positive appraisals of relationship quality. The perceived practical and emotional support from networks (relatives, friends, housekeepers) was highlighted.

One of the best things my two children did was, on Carnival Tuesday, one staying with my wife and the other taking me to the cinema. (spouse)

I often lose temper and raise my voice with Mum… But our maid of many years is very funny and helps me to calm down and to mark the positive side. She helps taking the pressure off, like in those pressure cookers. (adult child)

A few participants gained valuable practical support from friends or colleagues experiencing similar situations.

I had a colleague whose mother had dementia. Proximity, talking, helped a lot because there were things that I shared with her, and she helped me noticing a pattern I hadn’t noticed. (adult child)

Where support was absent, other carers expressed difficulties embracing the caregiving role all alone. Sibling relationships were often outlined, mostly as supportive but sometimes as examples of lack of family solidarity.

I just don’t understand how my brother doesn’t care, when our mother always cared for him. (adult child).

Family is supposed to help, isn’t it? Not mine, though. Instead of helping they just undermine my efforts. When they visit us, there is always a conflict. And I’m sure my husband notices that. It’s difficult for me and for him. (spouse)

People with dementia and carers identified the benefits of talking with professionals who could act as impartial and active listeners. They valued being able to talk with someone neutral, as home care professionals or psychiatrists, while they were reluctant to share their feelings with the informal networks to avoid burdening them. Many also considered that their relatives and friends were either too emotionally attached, tending to offer advice or emphasise their own experiences instead of empathising, or lacked knowledge about dementia and understanding. At 3-year, some carers regretted having postponed formal support requests.

I finally found someone (psychiatrist) who was going to listen to what I had to say. (person with dementia)

The turning point for me, as the main carer, but also for the entire family, was attending altogether that Alzheimer’s café session. For the first time, I felt understood and appreciated by my relatives. (spouse)

For most carers, better previous relationships were also linked to more positive assessments of current relationship aspects. Conversely, those few who perceived worse relationship quality prior to onset of dementia were more inclined to stress the negative aspects of caregiving.

We had a very good relationship, and this helps a lot now. When it comes to such critical moments, if the relationship was already bad, it gets much worse. (adult child)

Some carers reported past experiences with other diseases as something that helped dealing with dementia. However, they also acknowledged differences between both situations.

My wife had cancer some years ago and, together, we were able to deal with it. That helped me a lot, knowing it is possible to overcome challenges. The big difference is that with cancer there is hope and there is an end in sight. (spouse)

## Discussion

We studied a sample of community-dwelling people with middle stage dementia and their family carers over three years, focusing on relationship quality domains.

Dementia impacts on relationships, interfering with specific aspects of their quality, such as ‘communication’, ‘closeness’, ‘similarity of views’, ‘shared activities’ and ‘generally getting along’ (overall satisfaction). However, quantitative and qualitative findings showed that some of these aspects remain intact or even improve after an initial decline. Maintaining a sense of mutuality (‘closeness’) probably protected the person with dementia from institutionalisation. By using mixed methods, we clarified that quantitative and qualitative findings may converge or not.

### Convergence between quantitative and qualitative findings

Combining the two methods demonstrated that not all domains of relationship quality are affected in the same way, that changes occur at different stages, and that relationship appraisals of persons with dementia and their carers tend to differ.

The ‘closeness’ domain was consistently altered, from carers’ perspectives, over the three years. There was a significant decline in ‘closeness’ scores, especially from 12 to 36 months, in line with other longitudinal studies (Bruinsma et al., 2020; Clare et al., 2012). Therefore, a cross-sectional approach of the impact of dementia on relationship domains may be misleading: ‘closeness’ may be perceived positively, as in our baseline assessment of the larger EU-Actifcare cohort (Marques et al., 2019), but worsen longitudinally. Our qualitative findings suggest that people with dementia and carers report stronger emotional bonds at earlier stages of dementia, ‘closeness’ being challenged thereafter. This aligns with a qualitative study wherein, despite attempts to sustain a ‘nurturative relational context’, some dyads grew apart over five years (Hellstrom et al., 2007). Emotional detachment may occur as a coping mechanism when carers face stressful situations (Fauth et al., 2012). Alternatively, depression might impair carers’ ability to feel affectionate warmth (Mortazavizadeh et al., 2020). Other qualitative data reinforced that changes in ‘closeness’ are important to assess perseverance time (i.e. how long can carers still provide their current care) (Kraijo et al., 2014) and proneness to institutionalise. Overall, a lasting sense of mutuality in the relationship is key to care commitment (Hirschfeld, 1983), reflecting the importance of the relationship quality before the dementia onset.

Both quantitative and qualitative approaches also showed that changes in relationship quality occurred at different stages of dementia, while aspects of the relationship remained intact or even improved after initial decline.

Regarding ‘communication’, there was a non-statistically decline trend over the three years (with carers consistently rating this domain lower than their relatives) while the qualitative data also showed how communication was challenged. However, some positive appraisals of ‘communication’ were also identified. This was the case for maintaining shared activities, suggesting a potential for change, even throughout the later stages. The corresponding qualitative analyses showed that most carers coped adaptively underlining the relevance of longer follow-ups. This may indicate the difficulty of preserving relationships as dementia progresses, and the possible protective effect of distancing, for the carer (Woods et al., 2003). Language impairments and behavioural problems (for example, apathy) were associated with perceived deterioration in ‘communication’, as previously reported (de Vugt et al., 2003; Wong et al., 2020). Additionally, some carers improved their ‘communication’ during follow-up, through family or formal support. Therefore, ‘communication’ is potentially modifiable when behaviour problems, especially apathy, occur, and can be further enhanced by therapy that focuses on improving skills and coping with language and communication difficulties in dyads (Olthof-Nefkens et al., 2022). A one-year follow-up of people with Alzheimer’s disease and their carers suggested that relationships were ‘dynamic co-constructions’, built from daily interactions (Graham & Bassett, 2006). This on-going process provides a basis for family interventions.

Finally, both approaches also showed that relationship changes may be perceived differently by each member of the dyad: persons with dementia tend to appraise relationship quality more favourably than carers, aligning with previous findings (Ascher et al., 2010; Clare et al., 2012; Mortazavizadeh et al., 2020; Rippon et al., 2020; Spector et al., 2016; Wright, 1991). From carers’ perspectives, ‘closeness’ and ‘sharing activities’ were affected over the three years. People with dementia perceived deterioration mainly on ‘similarity of views’ and ‘generally getting along’. Previous studies demonstrated the dynamic nature of dyadic relationships within the ‘personhood’ context and discrepancies between persons with dementia’ preferences and carers’ perceptions of these preferences (Davies & Gregory, 2016; Woods, 2001). Such discrepancies often imply relational tension. Considering the ‘person’ (Kitwood, 1993) remains crucial to identify psychosocial factors amenable to intervention (Woods, 2001) while the recent literature on ‘couplehood’ (Stedje et al., 2023) emphasises the systemic perspective.

### Divergence between quantitative and qualitative findings

Qualitative findings allowed us to understand that the experience of ‘mixed feelings’ can be harder to manage than negative feelings alone (Losada et al., 2017). Combined with non-acceptance of ambivalence as normal in dementia caregiving, this can provoke anxiety and guilt (Cheng, 2017). Moreover, maladaptive coping, as avoidance of negative emotions, potentiates distress. Our findings echo reports that ambivalence correlates with caregiving stressors, contributing to carers’ depressive and anxious symptoms (Losada et al., 2017). Contrariwise, caring by free choice has been strongly associated with carers’ well-being (Al-Janabi et al., 2018; Oyebode & Parveen, 2019). Given the difficulties in managing contradictory feelings and associated distress, this calls for greater focus on carers’ emotion regulation and problem-solving skills when facing role conflicts or dilemmas about care decisions.

Not evident from the quantitative results, qualitative findings also pinpointed perceived social support and receiving formal support as positive appraisals of relationship quality. Practical and emotional support was especially protective for carers experiencing changes in living situations and social restrictions affecting carer roles within the larger family. This aligns with evidence that family cohesion and access to services are important (Yu et al., 2017). However, consistently with others (Cheng et al., 2013, 2022), we also showed that some carers felt disappointed when expectations of family support were unmet. Although there is usually one member of the family that takes the lead, caregiving systemically impacts the whole family (Gonçalves-Pereira et al., 2017). When families face chronic health conditions, the system must adapt dynamically. Research on wider social support networks, not just the main ‘carer’, is crucial in dementia caregiving (Gonçalves-Pereira et al., 2020).

Our results also showed that people seek formal help at different times and for different reasons, and that interventions should be tailored to family needs and resources (Moniz-Cook & Rewston, 2020). The Actifcare project highlighted that gradual build-up in care use (e.g., starting with domestic support; meals on wheels) improves access to other services as it contributes to overcome reluctance to accept help (Kerpershoek et al., 2019).

Even in a south-European country with strong family values, emerging challenges (i.e., global ageing, difficulties to accommodate the caregiving role) are jeopardising the family as the most powerful resource in dementia. Portugal will soon have one of the highest percentages of older people in the world, and the societal burden of dementia is indisputable (Gonçalves-Pereira et al., 2021; Nichols et al., 2022). The present study adds to our previous analysis of access and use of community services (Gonçalves-Pereira et al., 2019), highlighting problems to meet the needs and leverage the resources of these Portuguese dyads.

### Implications for research and practice

In this study, mixed methods broadened our horizons, moving away from reductionist dichotomous conceptualizations of relationship quality. They also elicited alternating or concurrent negative and positive, ambivalent feelings toward their relatives, as part of interviewees’ understandable experiences and yielded a richer picture of relationship quality appraisals. Using longitudinal designs was crucial to understand relationship changes, as clinical and social scenarios also change. Finally, combining individual and joint interviews of persons with dementia and carers, provided more holistic views on subjects and the couple. Individually, in-depth explorations and self-disclosure may be easier, not least because of persons with dementia’ cognitive or language difficulties, or carers’ avoidance of hot topics in front of their vulnerable loved ones. Joint interviews allowed the dyad members to facilitate each other disclosures (in our study, these dynamics apparently elicited positive appraisals and experiences of gratitude), and people with dementia may sometimes feel more at ease. This may inform future research.

There are also clinical implications. While some carer interventions may also impact on persons with dementia (Cheng et al., 2022), our work supports the need to consider couple and family interventions more frequently. Knowledge of dyadic relationships is critical to detect care needs. Social and healthcare professionals should attend to modifiable relationship domains, such as ‘communication’ or ‘shared activities’. Compromising relationships also compromises quality of care and triggers negative feelings, potentiating negative outcomes. Poor relationship quality may also lead to premature, unnecessary institutionalisation (Spruytte et al., 2001). Interventions to change relationship quality overall are usually difficult, lengthy, and costly (Cheng et al., 2019; Quinn et al., 2016). Instead, interventions tailored to specific relationship aspects may prove feasible, reinforcing what is right and not only mending what is wrong. Timely focus on relationship issues could identify those at risk to provide additional support.

### Strengths and limitations

To our best knowledge, this study is unique in its mixed method approach to relationship quality in dementia. We collected extensive quantitative and qualitative data from both dyad members over a relatively prolonged follow-up. Our findings were also enriched by interviewing dyad members both separately and jointly.

Limitations include the relatively small size of our convenience sample. Results may reflect family caregiving arrangements in a south-European community-dwelling context, limiting generalisability further. Finally, by using a convergent mixed methods design, contradictions between quantitative and qualitative data may require the collection of additional data, despite suggesting new insights on their own.

## Conclusion

Our study contributes to the growing body of research on relationship quality in dementia. Conducted prior to the COVID-19 pandemic, the role of relationship quality now seems even more apparent. Pandemic-related restrictions on informal and formal support led to increasing social isolation and carer stress (Giebel et al., 2020) with a higher risk of family conflicts and carer thoughts of giving up (Losada et al., 2022).

This calls for timely psychosocial interventions to improve health-related outcomes and quality of life, enhancing interactions within families. Interventions should tackle modifiable aspects of the relationship, meeting specific needs and cherishing the resources of different dyads and families as dementia progresses.
